# Inferring Passenger Denial Behavior of Taxi Drivers from Large-Scale Taxi Traces

**DOI:** 10.1371/journal.pone.0165597

**Published:** 2016-11-03

**Authors:** Sihai Zhang, Zhiyang Wang

**Affiliations:** Key Laboratory of Wireless-Optical Communications, Chinese Academy of Sciences, University of Science and Technology of China, Hefei, Anhui, 230017, China; West Virginia University, UNITED STATES

## Abstract

How to understand individual human actions is a fundamental question to modern science, which drives and incurs many social, technological, racial, religious and economic phenomena. Human dynamics tries to reveal the temporal pattern and internal mechanism of human actions in letter or electronic communications, from the perspective of continuous interactions among friends or acquaintances. For interactions between stranger to stranger, taxi industry provide fruitful phenomina and evidence to investigate the action decisions. In fact, one striking disturbing events commonly reported in taxi industry is passenger refusing or denial, whose reasons vary, including skin color, blind passenger, being a foreigner or too close destination, religion reasons and anti specific nationality, so that complaints about taxi passenger refusing have to be concerned and processed carefully by local governments. But more universal factors for this phenomena are of great significance, which might be fulfilled by big data research to obtain novel insights in this question. In this paper, we demonstrate the big data analytics application in revealing novel insights from massive taxi trace data, which, for the first time, validates the passengers denial in taxi industry and estimates the denial ratio in Beijing city. We first quantify the income differentiation facts among taxi drivers. Then we find out that choosing the drop-off places also contributes to the high income for taxi drivers, compared to the previous explanation of mobility intelligence. Moreover, we propose the pick-up, drop-off and grid diversity concepts and related diversity analysis suggest that, high income taxi drivers will deny passengers in some situations, so as to choose the passengers’ destination they prefer. Finally we design an estimation method for denial ratio and infer that high income taxi drivers will deny passengers with 8.52% likelihood in Beijing. Our work exhibits the power of big data analysis in revealing some dark side investigation.

## 1 Introduction

The deluge of data is revolutionizing our world. Properly collected and intelligently processed big data are able to provide significant insights in various application fields. For example, statistic inferences based on big data analytics have shown their powers in biology [1–3], medical science [4, 5], sociology [6–8], geography [9], zoology [10], intelligent transportation [11, 12], to name a few. Among all these, traffic behavior belongs to the scope of Cyber-Physical Systems(CPS), which aims to answer the questions from deeply intertwined physical and cyber components [13], and two examples are unsignalized intersections with heterogeneous urban traffic [14] and empirical investigation on driver behavior [15]. This paper provides a case study of big data analytics in the taxi industry. Since taxis bear a substantial amount of transportation burden and supply flexible ways of travelling in urban areas, investigation of the taxi industry is of particular interest to public administration. Researches in this area include safety and health assessments of taxi drivers [16][17], labor supply issues [18], choices of commuting modes [19], etc. However, these works often rely on simplified models and lack of supports from large-scale real data. In recent years, the development of modern information technologies has enabled the collection of large-scale trace data of taxis for the use of surveillance and management. They also provide abundant opportunities for big data research. Refuel events of taxi drivers are investigated in [20] so as to estimate citywide petrol consumptions. Malicious detour behaviors are recognized, which protects passengers from paying unjustified expenses [21].

However, one striking disturbing events commonly reported in taxi industry is passenger refusing or denial, whose reasons vary, including skin color [22], blind passenger [23], being a foreigner or too close destination [24], religion reasons [25] and anti specific nationality [26], so that complaints about taxi passenger refusing have to be concerned and processed carefully by local governments. In addition, through analyzing trace data, one can also identify those experienced taxi drivers, learn their driving patterns, and improve the others’ driving qualities [27]. Data analysis is also able to help taxi drivers find their potential passengers in a more efficient way [28]. The experience and knowledge of high income taxi drivers are analyzed and minded, to reveal the inconspicuous service strategy [29, 30], which is coined as Mobility Intelligence. These works imply that income differentiation exist in taxi drivers, and high income taxi drivers is explained to earn more income because they can choose proper waiting areas to pick up more passengers based on their intelligence. In this paper, we inspect this issue again, and focus on answering the following questions: (1) Will this preferential pick-up strategy surely increase taxi drivers’ income? (2) Are there any other factors that contribute to the high income of certain taxi drivers? The answers to the above two questions are No and Yes, respectively.

Based on the complete trace information for taxis supplied by one Chinese Investigation Agency, we study the income differentiation, pick-up diversity, drop-off diversity and grid diversity in all taxi drivers. Our works are performed at both the individual and group levels. To ensure better statistics, the top 3,590 taxis with the largest number of served passengers are chosen as our data sample, each having more than 932 single trips and millions of GPS report records. We propose a bottom-up approach to investigate individual taxi driver’s pick-up behavior: (1) finding out the actual income of each taxi driver, (2) grouping taxi drivers into different income level with proposed diversity concepts, and (3) understanding the pick-up and drop-off patterns for each group. We filter out four groups of taxi drivers according to their pick-up and drop-off diversity, and each group comprising drivers with High income(525 drivers), Medium high(542 drivers), medium low(510 drivers) and low(516 drivers), respectively. We demonstrate that the four groups exhibit different pick-up and drop-off patterns and that the high income drivers exhibit passenger denial behaviors (e.g., they refuse passengers after knowing their destinations.).

In summary, we have two important findings in this paper. (1) The so called mobility intelligence do not necessarily increase the income of taxi drivers, unless they choose the proper waiting areas. Here, high income and low income taxi drivers are two opposite examples. (2) High income taxi driver are exposed to deny passengers after knowing their destinations and the estimated denial rate of high income taxis is 8.52%.

## 2 Materials and Methods

### 2.1 Data Description

The data was collected from 12,657 taxis equipped with GPS devices during a period of two months (from November 1st to December 31st, 2012). The sampling interval ranges from 10 seconds to 60 seconds, depending on the settings of different taxis. There are totally around 2 billion rows of records. The data fields are described in Table 1. Note that under the permissions of the taxi drivers, the data was collected by that Agency and allowed to be used in the authors’ scientific research. The data has been anonymized and contains no personal information.

**Table 1:** Data Fields of Taxi Traces.

Field	Description
TAXI_ID	Unique ID of the taxis
OPERATION STATUS	Status of the taxi.
	0: vacant
	1: occupied
	2: others
EVENT TRIGGER	Status transition of the taxi.
	0: from occupied to vacant
	1: from vacant to occupied
	2: others
TIME STAMP	Time of the record(second)
LONGITUDE	Longitude of the taxi
LATITUDE	Latitude of the taxi
SPEED	Instant speed(kilometer/hour)(Range: [0, 255])
ORIENTATION	Taxi facing clockwise orientation(degree)
	Range: [0, 360], 0 stands for the north.
GPS STATUS	status of the GPS device.
	0: recorded GPS data is correct;
	1: recorded GPS data is wrong

### 2.2 Data Preprocessing

In the collected raw data, some of the records contain errors. Hence, prior to data analysis we perform preprocessing as follows. First, we classify the records according to the TAXI_IDs and remove duplicated records associated with every TAXI_ID. Second, we move the TAXI IDs that associate with less than 500 records. Third, we remove erroneous records that have impossible speeds (beyond the range of [0, 120]), wrong locations (beyond the area of [115.4,117.6] in the east longitude and [39.4,41.1] in the north latitude) and wrong times (beyond the range from November 1st to December 31st, 2012). After these steps, we prepare a preliminary dataset containing 12,053 taxis.

From the preliminary dataset, we further delete those TAXI_IDs satisfying both of the following conditions: (1) its daily gross income is always close to 0; (2) its average daily working time exceeds 12 hours. Notice that the daily gross income and the daily working time can both be estimated from the records, as we will show in Section 2.5 and Section 2.6, respectively. We believe that these taxis signed long-term contracts with their passengers, and are hence beyond the scope of our investigation. Then we have a refined dataset from 8,846 taxis for data analysis, which is called DATASET #1. Within the refined dataset, 3,590 taxis that work every day from November 1st to December 31st, 2012, are of particular interest to us and called DATASET #2.

### 2.3 Defining Trips of Passengers

The status of a taxi changes from vacant to occupied when a passenger starts his/her trip, keeps to be occupied all along the trip, and changes from occupied to vacant when the trip ends. Therefore, from the dataset we find trips based on the following three conditions: (i) they start with EVENT TRIGGER = 1 (status changing from vacant to occupied); (ii) they end with EVENT TRIGGER = 0 (status changing from occupied to vacant); (iii) between the starting and the ending points, OPERATION STATUS = 1 (occupied). We extract a series of records for each trip containing TIME STAMP, LONGITUDE, LATITUDE, SPEED, ORIENTATION, and GPS STATUS. From these records, we are able to calculate the distance, income, and gas consumption of each trip.

### 2.4 Calculating Driving Distances

**Fig 1:**
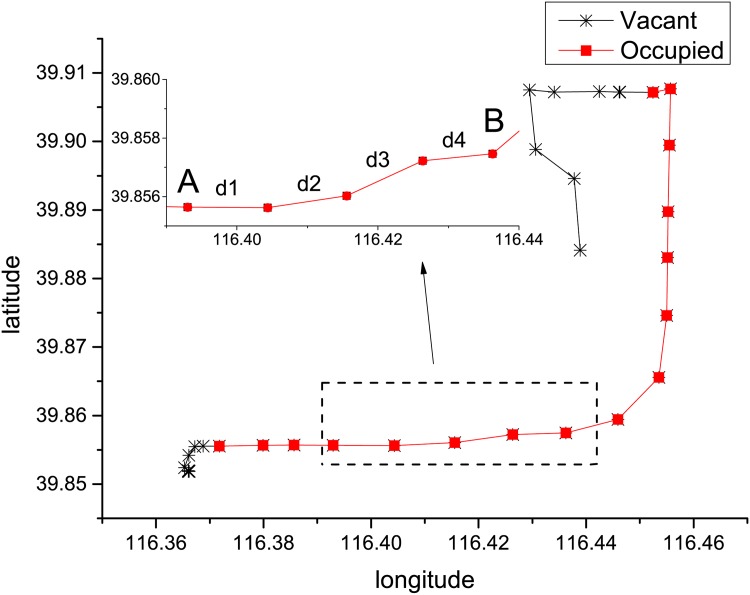
A route plotted by a taxi’s trace data. Each point denotes one reported GPS data with longitude and latitude information. The line segment between two consecutive points is approximately considered to be the actual driving route of the taxi. Note that *d*_*i*_ is the distance between two consecutive points calculated by above Formula (1) in this subsection.

Consider two points given by two consecutive records within a trip. Suppose that their longitudes are *long*_1_ and *long*_2_, while latitudes are *lat*_1_ and *lat*_2_. According to the spherical law of cosines, the distance *d* between the two points can be calculated based on haversine formula [31]:

$$\begin{array}{c} d=\cos^{-1}\left(\sin\left(lat_{1}\right)\sin\left(lat_{2}\right)+\cos\left(lat_{1}\right)\cos\left(lat_{2}\right)\cos\left(long_{1}-long_{2}\right)\right)R, \end{array}$$ (1)

where *R* is the radius of the earth. We approximate the total distance of a trip by summing up the distances between all pairs of the consecutive points along the trip. As an example, Fig 1 shows a route of a taxi and the way of calculating the driving distance.

### 2.5 Estimating Incomes

The taxi charge standards of Beijing in 2012 are as follows, based on which, we are able to calculate the charge of each trip, and hence estimate the gross income of each taxi driver.

1. Basic charge: 10 CNY for the first three kilometers, and 2 CNY for each additional kilometer during the day shift (5:00AM–22:59PM); 11 CNY for the first three kilometers, and 2.4 CNY for each additional kilometer during the night shift (11:00PM–4:59AM).
2. Charge for low speed driving and waiting: when an occupied taxi runs under the speed of 12 kilometer/hour or stops due to traffic jams or traffic lights, the time will be accumulated and the charge is 0.4 CNY per minute during the day shift and 0.48 CNY per minute during the night shift.
3. Charge for empty returning: when a trip exceeds 15 kilometers the passenger pays 50% extra fee, except that the line distance between the starting and the ending points is less than 2 kilometers.

Next we estimate the net incomes. The expenses are composed of two parts: costs of gas consumptions and fees paying to taxi companies, including management fees and taxes. According to the regulations of the taxi companies, every taxi driver pays a fee of 8,290 CNY per month.

**Fig 2:**
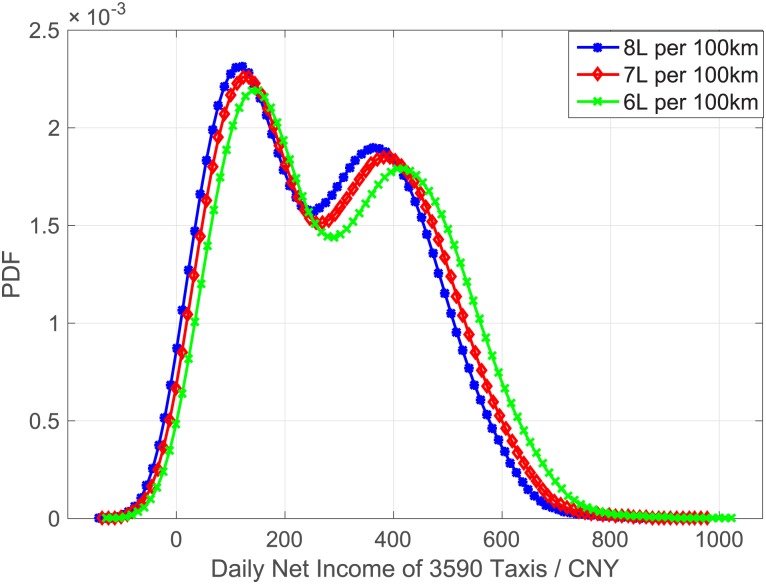
Daily Net Income comparison for different gas consumption = 6,7,8 liter/100km.

As for the costs of gas consumptions, we use popular rough estimation that taxis will cost V liter gas per hundred kilometers, neglecting other factors such as road situation, traffic status, number of passengers, season shift and etc. According to our common knowledge, gas consumption for taxis are around 8 liter/100km. In addition, we tested three parameters to compare the difference of income estimation using different gas consumptions, as presented in Fig 2, and found that, different gas consumption parameters hardly had influence on the income grouping. So in this paper, V is considered as 8 liter/100km and used in the following related analysis.

This volume multiplying the gas price (7.81 CNY per liter in Beijing from November 1st to December 31st 2012), is the taxi’s cost of gas consumption at that day.

### 2.6 Calculating Working Times

**Fig 3:**
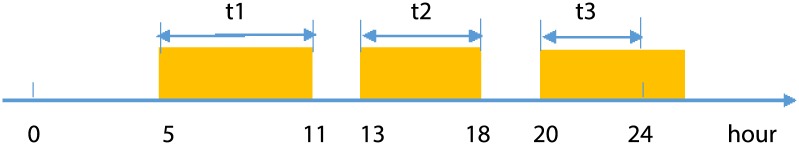
The yellow periods of time denote the taxi’s working time, and we only take the time period in one day into account, so the part after *t*3 that passes 24 o’clock will not be cumulated into that day’s working time.

We define that a taxi is working by the following rules. First, if its OPERATION STATUS is 1(occupied), then the taxi is working. Second, if its OPERATION STATUS is 0 (vacant) but its instant speed is not 0 or its location is different from the one in the previous record, then we know that the taxi is driving towards somewhere. For the latter case, the taxi is still working though it does not carry any passenger. Based on these two rules, we can calculate the working time of every taxi in every day. Fig 3 gives an example where the taxi is working in the periods with lengths *t*_1_, *t*_2_ and *t*_3_, all within the range from 0:00AM to 23:59PM, and the overall working time is *t*_1_ + *t*_2_ + *t*_3_.

### 2.7 Separating Geographical Grids

We divide the city of Beijing into adjacent grids with the size of 300*m* × 300*m*, not considering the existence of city roads, functional districts, forbidden areas, undeveloped regions, etc. Then we get 21,312 grids in total. Our focus is on the central area of the city (longitude within [116.198,116.7125] and latitude within [39.75,40.150]), containing Dongcheng, Xicheng, Haidian, Chaoyang, and Shunyi Districts. This central area contains 148 × 144 = 21,312 grids. We analyze the events of passenger pick-ups and drop-offs within these grids.

## 3 Results

**Fig 4:**
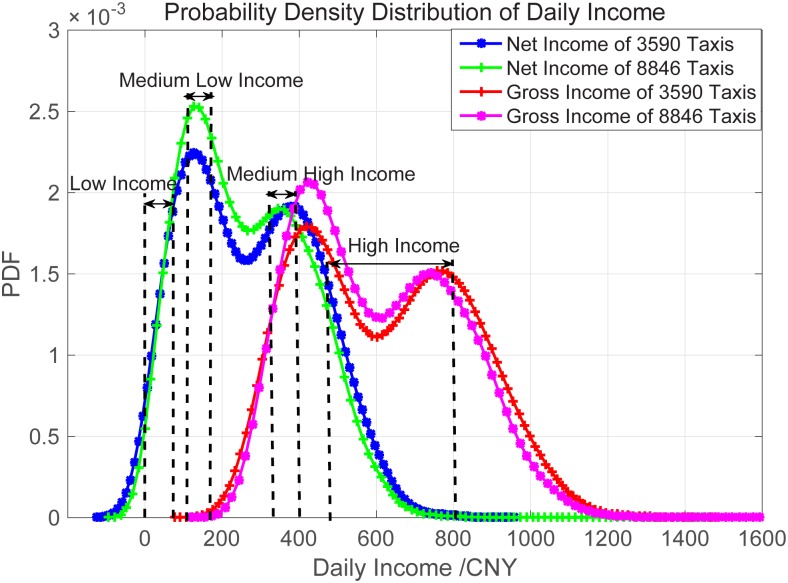
The probability density distributions of taxis income in Beijing during Nov.- Dec. 2012. (a) The magenta curve illustrates the net income of all 8846 taxis, and the blue square curve illustrates that of the selected 3590 taxis which operate every day during two months. (b) The number of selected taxis in Low Income, Medium Low Income, Medium High Income and High Income are 525, 542, 510, and 516, respectively. (c) The average daily income of taxis in each group are, 532.387, 360.985, 139.124, and 51.7747 CNY, respectively. (d) The shift from Net Income to Gross Income (The red diamond curve) denotes the taxi daily cost, which is inferred to be 337.5 CNY for each Taxi.

Having the taxis’ daily net and gross incomes at hand, we plot their probability distribution functions (PDFs) in Fig 4. The two datasets, DATASET #2 (with 8,846 taxis) and DATASET #1 (with 3,590 taxis that work every day), have similar PDFs of both net and gross incomes. It is obvious that the daily incomes of the taxi drivers differ significantly. In the existing research, this phenomenon has been explained as the consequence of mobility intelligence, meaning that some taxi drivers deliberately choose their strategies to earn more profits. That is, according to their experiences, high income taxi drivers are often intelligent enough to choose proper areas to wait for and pick up more passengers.

In this paper, we investigate the in-depth reason for the differentiation of incomes, and obtain several important results. In subsection 3.1, three main factors, working time, passenger load, distance per trip, are discussed, but verified not correlated with this income differentiation. In subsection 3.2, grid diversity analysis reveals that, high income taxis prefers both waiting for and dropping passengers in their preferred limited areas, compared to other three groups. In subsection 3.3, by confirming the negative answer for the correlation between pick-up grids and drop-off grids, the passenger denial behavior of high income taxi drivers are confirmed. In addition, we estimate that high income drivers in this dataset will deny 8.52% passenger in average.

### 3.1 Grouping Taxis by Incomes

From DATASET #1, we arbitrarily pick four groups of taxis according to their daily gross incomes: High (from 460 to 800 CNY and with 516 taxis), Medium High (from 110 to 320 CNY and with 510 taxis), Medium Low (from 110 to 170 CNY and with 542 taxis), and Low (from 0 to 80 CNY and with 525 taxis). The average daily gross incomes of the four groups are 532.387, 360.985, 139.124, and 51.775 CNY, respectively.

Intuitively, there are three main factors that cause the huge difference in the daily incomes of the taxis: working time, passenger load, and distance per trip. Below we investigate their impacts on the daily incomes through analyzing the collected data.

**Fig 5:**
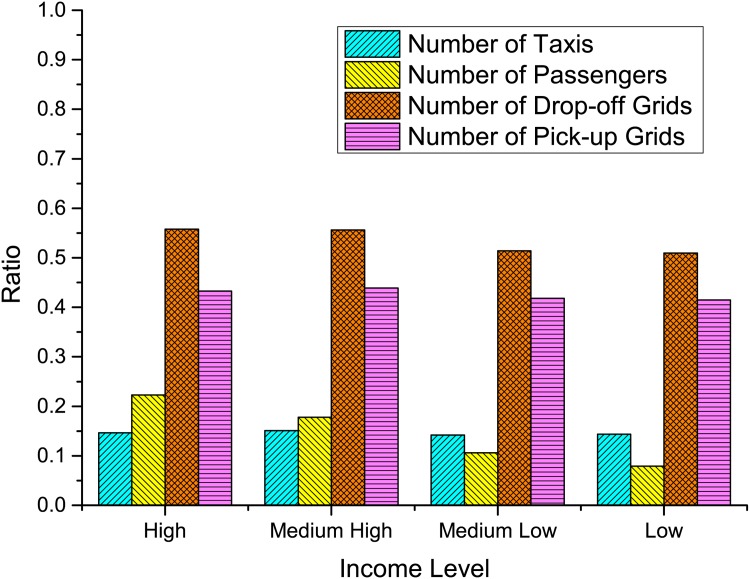
(a) This proportion statistics are based on the selected 3,590 taxis, among which, four income level taxi drivers are selected. (b) The proportions are taxi number, passenger number, of four income levels of taxi total number of taxis, total number of passengers served, total number of grids where drivers pick up and drop off the passengers. (c) The pick-up and drop-off grids covered by all these four income level taxis occupy about 41 and 54 percentage of all city grids, respectively.

**Fig 6:**
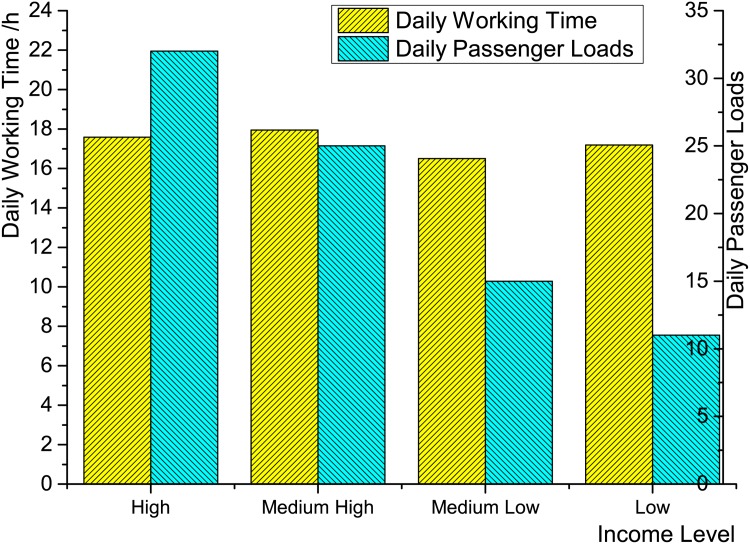
The average daily working time and served passengers of four groups of selected 3590 taxis.

**Fig 7:**
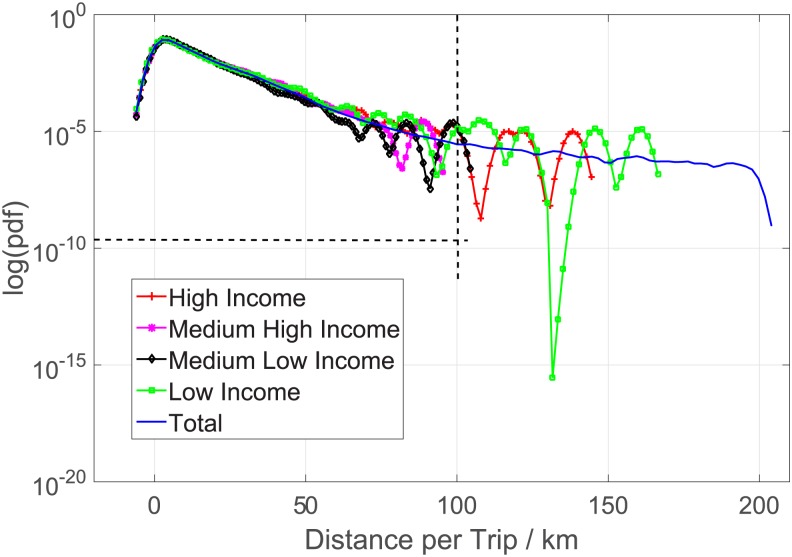
The probability distributions of the distance per trip.

**Fig 8:**
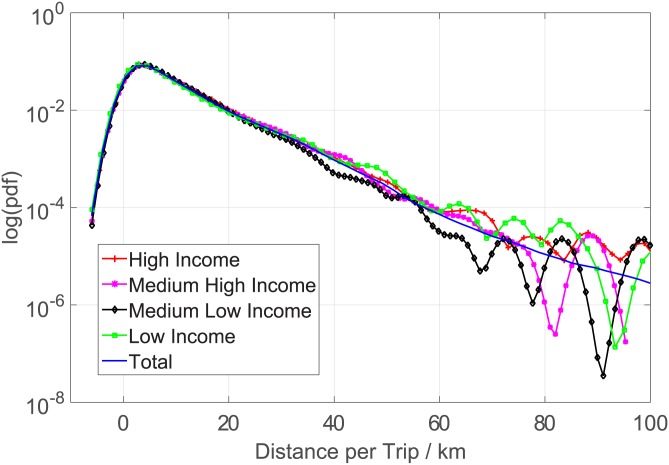
The probability distributions of the distance per trip shorter than 100km.

1. Working time. Fig 5 demonstrates that the average daily working times of the four groups of taxis are very similar. A taxi (often with two drivers serving day and evening shifts, respectively), no matter its daily income is high, medium high, medium low, or low, works around 16-18 hours every day. Therefore, working time is not a determining factor of income.
2. Passenger load. Fig 6 shows the average daily passenger load of the four groups of taxis. In average, a high-income taxi serves around 33 passengers everyday, while a low-income taxi serves only around 12. For medium high- or medium low-income taxis, the numbers are 25 and 15, respectively. This result provides us a strong evidence that the income is positively correlated with the working efficiency.
3. Distance per trip. Is a low-income taxi able to earn more money through driving more long-distance trips, though it serves less passengers everyday? Figs 7 and 8 gives a negative answer, shows that the average distances per trip are similar in, especially in the range of short distance (<20 kilometers).

The analysis on the dataset reveals that a high-income taxi has higher working efficiency than those in the other groups, which leads to extra profit. Now a natural question arises: what strategy does a high-income taxi adopt so that it is able to serve more passengers everyday? To answer this question, we proceed to investigate the pick-up and drop-off areas of the taxis.

### 3.2 Pick-up/Drop-off Diversity Analysis

We define the following notations for further analysis. Let $V_{i}^{p}$ be the number of grids where taxi *i* picked up passengers during the two months and $V_{i}^{d}$ be the number of grids where taxi *i* dropped off passengers. Considering all passengers who were picked up in grid *m*, denote $V_{m}^{g}$ as the number of grids where the drop-offs happened.

Define $x_{i}^{p}$ as the total number of taxi *i*’s pick-ups and $x_{im}^{p}$ as the number of those pick-ups occurring in grid *m*. Apparently, $x_{i}^{p}=\sum_{m}x_{im}^{p}$. Therefore, $p_{im}^{p}=x_{im}^{p}/x_{i}^{p}$ is the percentage that the pick-ups occurred in grid *m* for taxi *i*. Similarly, define $x_{i}^{d}$ as the total number of taxi *i*’s drop-offs and $x_{in}^{d}$ as the number of those drop-offs occurring in grid *n*. We have $x_{i}^{d}=\sum_{n}x_{in}^{d}$ and define $p_{in}^{d}=x_{in}^{d}/x_{i}^{d}$ as the percentage that the drop-offs occurred in grid *n* for taxi *i*.

Define $y_{m}^{g}$ as the total number of trips starting from grid *m* and $y_{mn}^{g}$ as the total number of trips from grid *m* to *n*; $y_{m}^{g}=\sum_{n}y_{mn}^{g}$. Define $p_{mn}^{g}$ as the percentage of the drop-offs in grid *n* among all the trips that start from grid *m*, namely, $p_{mn}^{g}=y_{mn}^{g}/y_{m}^{g}$.

We further define two indices that quantify the diversities of pick-ups and drop-offs from the perspective of every taxi. The first one is the diversity that the pick-ups of taxi *i* occurred over all the grids:

$$\begin{array}{c} Div_{i}^{p}=\frac{-\sum_{m}p_{im}^{p}log\left(p_{im}^{p}\right)}{log(V_{i}^{p})}. \end{array}$$

Recall that $p_{im}^{p}=x_{im}^{p}/x_{i}^{p}$ is the percentage that the pick-ups occurred in grid *m* for taxi *i*. Hence, the numerator $-\sum_{m}p_{im}^{p}log\left(p_{im}^{p}\right)$ denotes the entropy of the pick-up grids; the entropy is further normalized by the denominator $log(V_{i}^{p})$ where $V_{i}^{p}$ be the number of grids where taxi *i* picked up passengers. Observe that larger $Div_{i}^{p}$ means that the pick-up grids of taxi *i* are more diverse. Similarly, the second diversity characterizes the drop-offs of taxi *i* occurring over all the grids, as denoted by:

$$\begin{array}{c} Div_{i}^{d}=\frac{-\sum_{n}p_{in}^{d}log\left(p_{in}^{d}\right)}{log(V_{i}^{d})}. \end{array}$$

Larger $Div_{i}^{d}$ means that the drop-off grids of taxi *i* are more diverse.

We define another variable that quantifies the diversity of drop-offs from the perspective of every pick-up grid:

$$\begin{array}{c} Div_{m}^{g}=\frac{-\sum_{n}p_{mn}^{g}log\left(p_{mn}^{g}\right)}{log(V_{m}^{g})}. \end{array}$$

Here $p_{mn}^{g}$ is the percentage of the drop-offs in grid *n* among all the trips that start from grid *m* and $V_{m}^{g}$ is the number of grids where the drop-offs happened. Larger $Div_{m}^{g}$ means that the drop-off grids of the trips from the pick-up grid *m* are more diverse.

With particular note, when $V_{i}^{p}=1$ (taxi *i* only picked up passengers from one grid) we let $Div_{i}^{p}=0$. Similar rule applies to $Div_{i}^{d}=0$ when $V_{i}^{d}=1$ and $Div_{m}^{g}=0$ when $V_{m}^{g}=1$.

**Fig 9:**
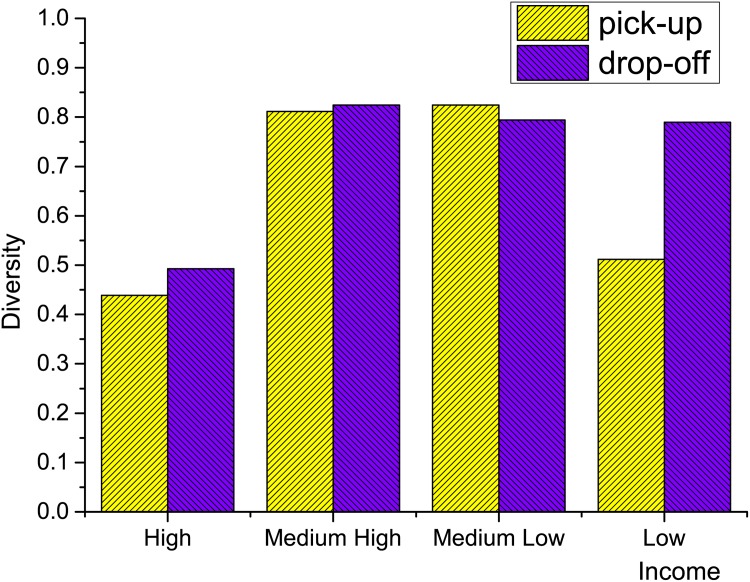
The pick-up and drop-off diversity of different income groups of taxis.

For the four groups of taxis with different incomes, Fig 9 shows their average pick-up and drop-off diversities. It is interesting to observe that the pick-up diversity of the high income taxis is around 0.44, which is significantly smaller than the medium high income taxis (0.80) and the medium low income taxis (0.81). For the low income taxis, this value is around 0.51. This fact shows that the two extreme groups, the high and low income taxis, prefer picking up passengers from less grids. More interestingly, the drop-off diversity of the high income taxis is around 0.50, which is not only significantly smaller than the medium high income taxis (0.81) and the medium low income taxis (0.79), but also far less than that of the low income taxis (0.79). Therefore, we can see that the high income taxis prefer dropping off passengers in less grids while the others do not.

**Fig 10:**
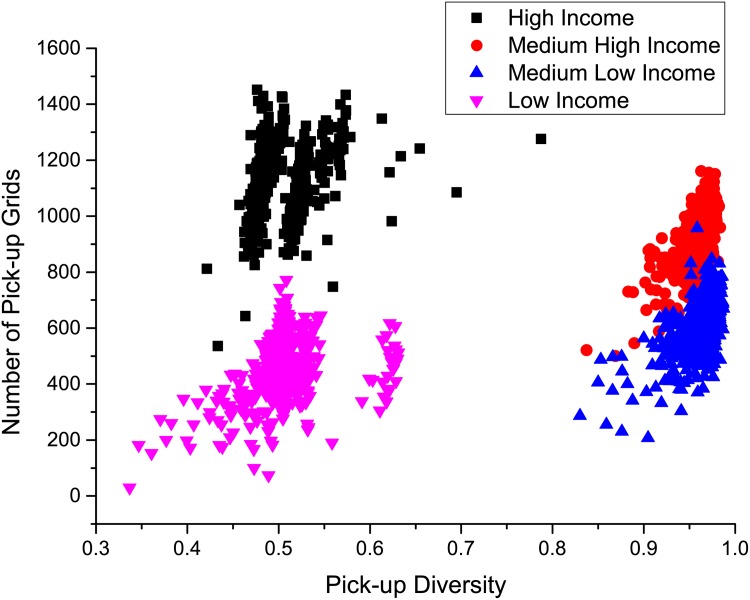
The number of picking up grids $N_{i}^{p}$ with respect to the pick-up diversity $Div_{i}^{p}$ of each taxi. Four clusters correspond to four income level taxis.

**Fig 11:**
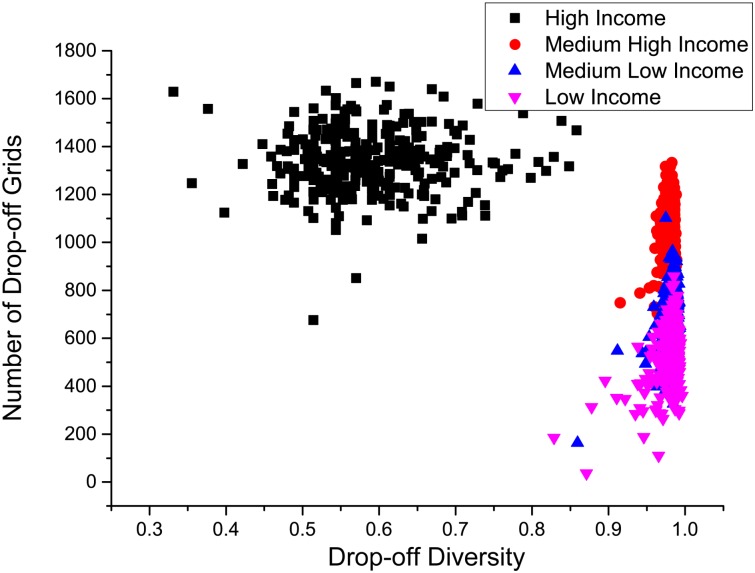
The number of dropping off grids $N_{i}^{d}$ with respect to the drop-off diversity $Div_{i}^{d}$ of each taxi.

These conclusions are further validated by Figs 10 and 11. Fig 10 shows the pick-up diversities of all the taxis versus the number of pick-up grids. Both high and low income taxis have small pick up diversities (centered at around 0.5), but the high income taxis have much larger numbers of pick-up grids than the low income taxis. It means that a high income taxi mainly picks up passengers from a limited number of grids, but works more efficiently than a low income taxi so that it is able to cover more grids. For the medium high and medium low income taxis, the pick up diversities are large (centered at around 0.95) and the medium high income taxis have slightly larger numbers of pick up grids than the medium low income taxis. Fig 11 depicts the drop-off diversities of the four group of the taxis. For the medium high income, medium low income, and low income taxis, their pick-up diversities are all close to 1, while the numbers of drop-off grids explain the difference of their efficiencies and hence incomes. The high income taxis are very efficient so that their numbers of drop-off grids are as high as 1000. However, the majority of the drop-offs happened at a limited number of grids, which causes the low drop-off diversities (mainly between 0.45 and 0.75).

### 3.3 Inferring Purposely Ignoring Passengers

From the analysis above, we can learn two rules of thumb of earning more money.

1. Rule 1: waiting for passengers in a limited number of areas (very likely, popular areas such as stadiums, shopping malls, and night clubs).
2. Rule 2: dropping off passengers also in a limited number of areas.

Note that Rule 1 itself can not simply guarantee high income; contrarily, it may lead to low income. Also, these two rules seem to be confusing: though it is easy to choose the pick-up grids, tt seems difficult to choose the drop-off grids so as to earn more money. One may ask: are there any pick-up grids whose trips end in a limited number of drop-off grids and correspond to high profits? Below we give a negative answer from the data analysis.

**Fig 12:**
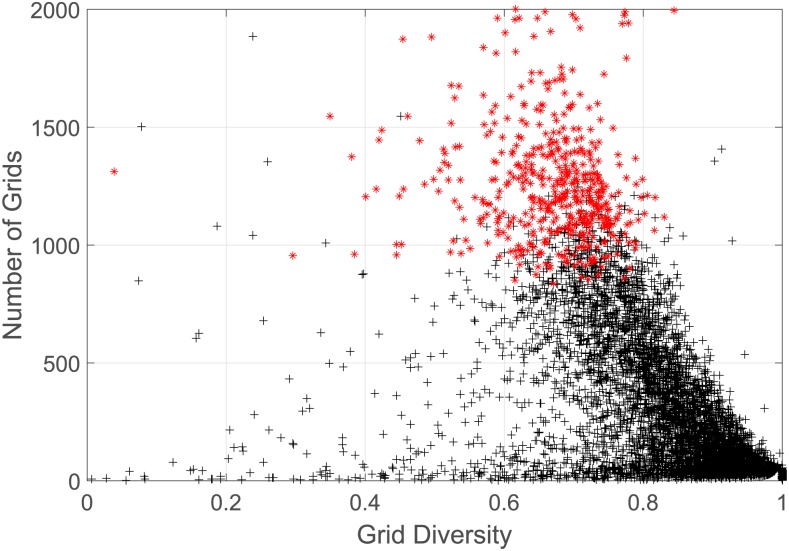
The number of dropping off grids $N_{m}^{g}$ with respect to the diversity of each grid $Div_{m}^{g}$, the black crosses represent each grid where pick-ups happen,totaling 12176, while the red asterisks represent the grids where high income taxis pick up more than 500 times, approximately 1 time per taxi, totaling 508.

Fig 12 presents the grid diversity of all pick-up grids. As we can see, most diversity are larger than 0.6, which means that the pick-up grids have quite low correlation with drop-off grids. Especially, the grids where high income taxis mainly pick up passengers mostly have the diversity in 0.6 ∼ 0.8.

These observations immediately arrive at an unfortunate conclusion that a high income taxi sometimes purposely ignores some passengers while prefers the others so that its destinations are concentrated in a limited number of drop-off areas, where it can pick up the next passenger more easily.

Below we propose an approach to estimate the rate of purposely ignoring passengers in the high income taxis. Assume that all the other taxis did not have such irregular behaviors—notice that this assumption undoubtedly leads to a conservative estimation.

As we have analyzed above, there truly exists passenger selection, or passenger denial in other words, when high income taxis drivers pick up passengers. That is the exact reason for their drop off diversity differences from medium and low income taxis. In this section, we further proposed a method to estimate the approximate passenger denial rate.

We assume that the drop-off grids caused by medium and low income taxis stand for the normal case which is of no passenger denial, thus the excessive amount of passengers that high income taxi drivers carry than other drivers, can be explained as the result of passenger denial. In other words, the existence of grids where high income taxis drop off more passengers means that, they reject some passengers whose destinations are not within their preferences.

**Fig 13:**
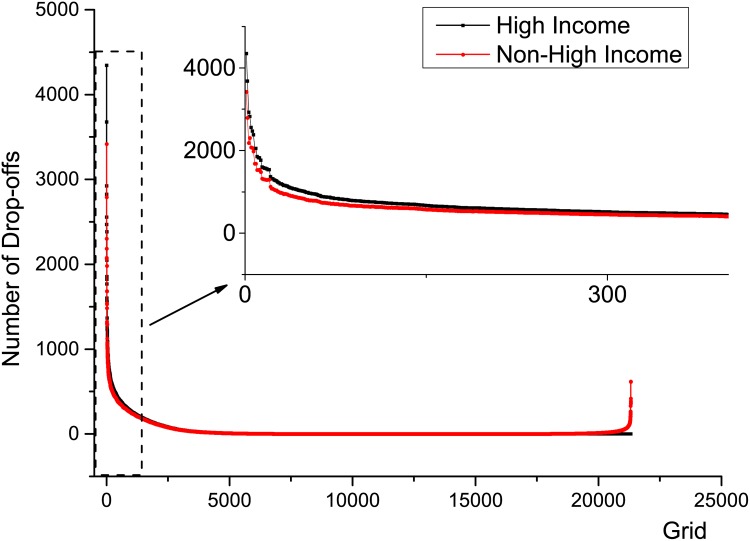
The drop-off distributions of high and non-high income taxis, the forepart is zoomed in to have a better view, from which we can see the non-high income taxis’ distribution is relatively more even.

Fig 13 presents the actual passenger amount carried by all high income and non-high income taxis in all grids, and the most busy 400 drop-off grids are also zoomed for details. So the denial rate can be estimated by:

$$\begin{array}{c} R_{d}=\frac{\sum_{i=1}^{k}H_{i}-nonH_{i}}{N} \end{array}$$

**Fig 14:**
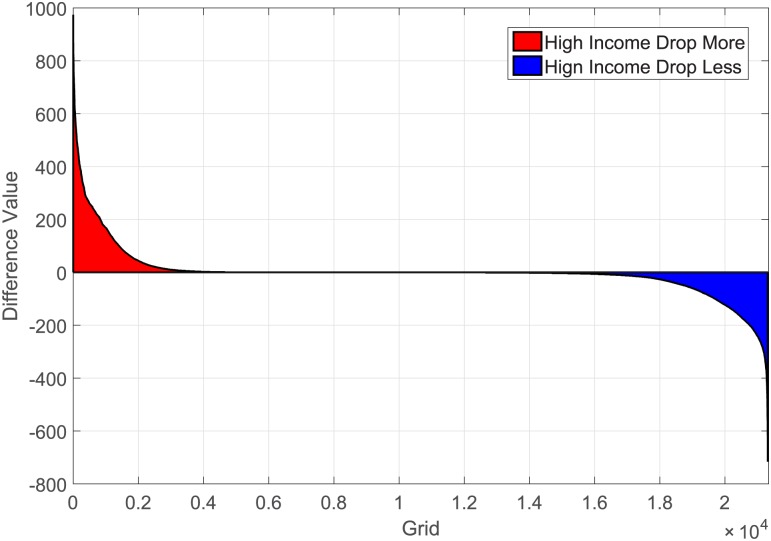
The difference value distribution between high and non-high income taxis, the blue part represents the passengers that the high income taxis refuse to take, and the red part is the high income’s preferential passengers, and obviously, the areas of these two parts are the same.

Here, suppose there are totaly *k* grids where the high income taxis drop off more passengers than non-high income taxis do. *H*_*i*_ is the number of drop-offs in grid *i* of high income taxis, similarly *nonH*_*i*_ is that of non-high income taxis. *N* is the total number of trips, or passengers, served in this situation. The difference value of high and non-high income taxis is plotted in Fig 14, the part below zero represents the passengers that high income drivers reject. Then we can calculate the denial rate of high income taxis is approximately 70288/825269 = 8.52%, where 70,288 passengers are estimated to be denied, in total 825,269 trips. According to the passenger loads in this case, the passenger percentage fulfilled by high income taxis is about 38.55%, so the estimated overall denial rate in all taxi drivers is about 3.28%.

## 4 Discussion

Through large-scale taxi trace data analysis, we confirm two facts that have been already revealed by previous work. First fact is income differentiation among taxi drivers. Second fact is, the mobility intelligence might be the reason for earning more money for taxi drivers. Those drivers who can choose proper pick-up areas will have more chances to serve possible passengers.

As to the high income taxi drivers, we gain the novel insight that, mobility intelligence is just one possible reason for their high income, and passenger denial also plays crucial role. The passenger denial estimation method considers only the high income drivers, but the actual denial rate in real world is reported much higher, which we believe is because the denial also happens in the medium and low income taxi drivers. But due the data limitation, estimation to this part is hard to perform.

Contrary to common belief, choosing pick-up areas does not necessarily lead to high income. Those examples of low income taxi drivers in this study illustrate that choosing wrong places is rather worse than randomly choosing, just as medium income taxi drivers do. Another interesting fruit obtained is that, it is believed that driving longer distance trip will bring higher income, but our results show that, the taxis drivers’ income do not have explicit correlation with the single trip’s distance.

The grid size is an important parameter in our research, which will affect the conclusions. But 300m × 300m is small enough to demonstrate the difference of pick-up and drop-off diversity among different group of taxi drivers, although we also believe that smaller size will lead to the same results. We argue that the pick-up and drop-off diversity concept proposed in this paper can be used to effectively classify taxis into different income level or even moral level, which has vital importance and tremendous potential in real applications, for example, taxi driver recognition.

We note that, all the computation tasks of this work need not consume huge computing time, which is helpful for building realtime diversity analytic system in the future. Suppose there are totally *N* gps records for *M* taxis, and the areas are divided into *K* × *K* square grids. Then for pick-up and drop-off diversity, the complexity is *O*(*N* × *K* × *K*), which is also the whole computation time complexity of this work, because other related computation operations, such as distance calculation, working time calculation, are all close to *O*(*N*) computation complexity.

The mobility diversity concept proposed in this paper have broad and significant potential applications in almost all mobility related areas. First, this diversity concept can be utilized as features or metrics for learning or recognizing mobile objects’ behavior, when using clustering, classification and machine learning techniques. Second, this diversity evaluation framework can be extended to distinguish different types of mobile objects in many other location based areas. For example, such analysis on hotel booking records can help understand the customers more accurately. We are convinced that this concept and framework will have much more practical case studies in the future.

Finally, fast developing information and communication technology can support the necessary data transmission and computation proposed in this paper. The coming 5G technology together with device-to-device(D2D) communications will enable the huge data collection and communications among vehicles and base stations or access points [32] and the reliable distributed and adaptive resource management for cognitive cloud vehicular networks can allow energy and computing-limited vehicle devices to utilize the available vehicle-to-infrastructure (V2I) WiFi connections for performing data offloading [33]. The wireless energy transfer can even enable the diversity computation in passive low-complexity devices such as sensors and wearable computing devices, if we want to analyze the location data generated or sensed from the above devices [34].

## 5 Conclusion

This research belongs to human dynamics topic, focusing on the distrss phenomena and internal mechanism for stranger to stranger interactions, which exists widely in almost every social areas of living life. We take taxi industry as example, but our methodology and framework may also contribute in other fields, which need further research and verification.

In summary, this paper has shown that leveraging large scale data set can enable simple or straightforward statistical inferring techniques to obtain significant insights on, in this case, taxi industry. The pick-up, drop-off and grid diversity concepts proposed and related diversity analysis done in this paper suggest that high income taxi drivers will deny passengers in some situations, so as to choose the passengers’ destination they prefer, which seems quite obvious to public, but has not been verified using realistic data set until our work. Thus, our work exhibits the power of big data analysis in revealing some dark side investigation for not only information system but also urban management and company regulations, which shed light for future investigation in other applications and domain.
