# Correction: Inferring Passenger Denial Behavior of Taxi Drivers from Large-Scale Taxi Traces

**DOI:** 10.1371/journal.pone.0171876

**Published:** 2017-02-06

**Authors:** Sihai Zhang, Zhiyang Wang

There is an incorrect figure referenced in the third paragraph of the “3.1 Grouping Taxis by Incomes” section of the Results. In the section titled “Working time”, “Fig 5” should be listed as “Fig 6”.

There is an incorrect figure referenced in the fourth paragraph of the “3.1 Grouping Taxis by Incomes” section of the Results. In the section titled “Passenger load”, “Fig 6” should be listed as “Fig 5”.
